# An ontologically founded architecture for information systems in clinical and epidemiological research

**DOI:** 10.1186/2041-1480-2-S4-S1

**Published:** 2011-08-09

**Authors:** Alexandr Uciteli, Silvia Groß, Sergej Kireyev, Heinrich Herre

**Affiliations:** 1 Institute for Medical Informatics, Statistics and Epidemiology (IMISE), University of Leipzig, Germany; 2 LIFE – Leipzig Research Center for Civilization Diseases, Universität Leipzig, Germany

## Abstract

This paper presents an ontologically founded basic architecture for information systems, which are intended to capture, represent, and maintain metadata for various domains of clinical and epidemiological research. Clinical trials exhibit an important basis for clinical research, and the accurate specification of metadata and their documentation and application in clinical and epidemiological study projects represents a significant expense in the project preparation and has a relevant impact on the value and quality of these studies.

An ontological foundation of an information system provides a semantic framework for the precise specification of those entities which are presented in this system. This semantic framework should be grounded, according to our approach, on a suitable top-level ontology. Such an ontological foundation leads to a deeper understanding of the entities of the domain under consideration, and provides a common unifying semantic basis, which supports the integration of data and the interoperability between different information systems.

The intended information systems will be applied to the field of clinical and epidemiological research and will provide, depending on the application context, a variety of functionalities. In the present paper, we focus on a basic architecture which might be common to all such information systems. The research, set forth in this paper, is included in a broader framework of clinical research and continues the work of the IMISE on these topics.

## Introduction

Clinical trials exhibit an important basis for clinical research. A clinical trial can be understood as a planned experiment which includes patients and is designed to gain insights into the etiology and progression of diseases, as well as to analyze new diagnostic and treatment procedures and, in particular, to test new drugs, [1], [2], [3].

Clinical and epidemiological studies can be divided into three stages, the stage of planning and design, the execution of the study, and, finally, the interpretation of the resulting data. Already the planning phase takes much time, due to the many aspects and tasks to be considered. The decision on the study design and the formulation of clinical questions and hypotheses needs an early involvement of various experts. Data managers create data entry forms for pseudonymous registration of records and specify the database concept. The specification of the data collection and documentation in clinical and epidemiological study projects represents a significant expense in the preparation of study projects. The planning and preparation of Case Report Forms (CRFs) of a study is carried out by data managers in collaboration with project managers, biometricians and computer scientists. A CRF describes a data entry form of a clinical trial, for instance, questionnaire, interview, laboratory or research protocol. It contains all the documentation features to be collected in a special data source process as part of a study.

The precise definition and semantically correct representation of the documentation features and study items has an important impact on the value and quality of these studies. It is an essential assumption to support the reuse of such features, to compare the data of different study projects, and to establish a common basis for the interpretation of the resulting data. It is however noticed that in clinical research these conditions are insufficiently realized. To solve this problem the study items must be captured and specified in a semantically correct way, and computationally presented such that they can be efficiently retrieved. However, there is no formal and well-established definition of the term *item*, which is the basic unit in clinical trials. In the CDISC Clinical Research Glossary (Clinical Data Interchange Standards Consortium [4], [5]), for example, the term item is defined as follows: "1. A representation of a clinical variable, fact, concept, or instruction in a manner suitable for communication, interpretation, or processing by humans or by automated means. ... 2. An individual question, statement, or task that is evaluated by the patient to address a particular concept to be measured by a PRO (patient-reported outcome) instrument."

The precise and complete specification of the notion of a study item which takes into consideration all the mentioned aspects set forth by CDISC, is a difficult task, and we believe that a broad, ontologically oriented view is useful to achieve a semantical correct representation of items, and to get a support to acquire, structure and retrieve the complex data in the field of clinical research. In the present paper we expound the basic structure of an architecture for information systems of this application domain. This architecture is grounded on a top-level ontology, since we defend the approach that a top-level ontology may provide a well-founded semantic basis for the considered entities, including items, metadata, and phenotypes.

There are several ISO standards (International Organization for Standardization [6]) providing systems of categories and relations as a framework for specifying data. We pursue the approach, that such standards must be taken into consideration to achieve an adequate metadata representation. On the other hand, these standards have, usually, an insufficient semantic basis. Hence, our ontological approach makes a further step towards a generic ontologically founded semantic framework to establish the semantics for such standardized systems, and thus, build a bridge between the rigor of formal ontology and the semantic vagueness of concept representation, found sometimes in ISO standards. In the framework of our project, we decided to take the ISO/IEC 11179 standard [7], [8] as our initial system, because it is tailored to the description and representation of metadata. This standard is specified by a generic system of categories and relations, which should usually be adapted to the particular domain under consideration. This adaption leads to the introduction of additional categories and relations.

The research, set forth in this paper, is included in a broader framework of clinical research and continues the work of the IMISE on these topics, see [9], [10], [11], [12], [13].

## Background and preliminaries

In this section we summarize and outline the relevant notions and methodological principles which are used throughout the paper.

### Basics of GFO

GFO (General Formal Ontology) is a top-level ontology being developed at the IMISE, university of Leipzig [14], [15]. In GFO, the entities of the world are classified into categories and individuals. Categories can be instantiated or predicated of other entities, whereas individuals do not satisfy these conditions [16], [17], [18]. Individuals are classified into concrete and abstract individuals. Concrete individuals are in space and time, whereas abstract individuals are independent of space and time. Concrete individuals are further classified with respect to the type of relation, which they have to space and time. They are categorized into *continuants*, *presentials* and *processes*. Another classification principle for concrete individuals, to be discussed in this section, pertains to the distinction between attributives and bearers.

Continuants persist through time and have a lifetime, being a time interval of non-zero duration, whereas *processes* happen in time and are said to have a temporal extension. A continuant exhibits at any time point of its lifetime a uniquely determined entity, called *presential*, which is wholly present at that time point. Examples of continuants are *this car*, *this ball*, *this tree*, *this kidney*, being persisting entities with a lifetime. Examples of presentials are *this car*, *this ball*, *this tree*, *this kidney*, any of them being wholly present at a certain *time point t.* Hence, the specification of a presential additionally requires a declaration of a time point.

Every process P has a temporal extension, which is a time interval of non-zero duration. These intervals are called in GFO chronoids. A chronoid is not understood as a set of its time points, but it is an individual entity *sui generis* (here, we rely on the philosophy of Franz Brentano [19]). In contrast to a presential, a process cannot be wholly present at a time point. Examples of processes are the *happening of a 100 M run during a time interval*, *and at a certain location with the runners as participants*, the *movement of a stone from location A to location B*, a *continuous change of the colour of a human face during a certain time interval*, a *surgical intervention at a particular temporal and spatial location*, or the *execution of a clinical trial*, *managed by a workflow*.

Continuants may change, because, on the one hand, they persist through time, on the other hand, they exhibit different properties at different time points of its lifetime. Hence, we hold that only persisting individuals may change. On the other hand, a process as a whole cannot change, but it may possess changes, or it may *be* a change. Hence, *to change* and *to have a change/to be a change* are different notions. In [15] basic types of changes, which a process may possess, are classified; we call such changes processual changes.

#### Integration of concrete individuals

A process has temporal parts, any of them is determined by taking a temporal part of the process’ temporal extension and restricting the original process to this subinterval. The relation temprestr(p,c,q) has the meaning, that p is a process, c a subinterval of the temporal extension of p, and q is that process, which is determined by restricting the process p to c. If we consider a time point of a process’ temporal extension, we allow the restriction of the process to this point. The relation tempbd(p,t,q) (q is temporal boundary of the process p at time point t) states, that p is a process, t is a time point of the temporal extension of p, and q is the result of restricting of p to t. q is called a process boundary of p at time point t. In GFO, the following axiom is stipulated.

#### Law of object-process integration

Let C be a continuant. Then there exists a uniquely determined process P, denoted by Proc(C), such that the presentials, exhibited by C at the time points of C’s lifetime, coincide with the process boundaries of P.

Assuming this integration law, we say that the continuant C supervenes on the process Proc(C), whose existence is assumed. We hold that a continuant C existentially depends, on the hand, on a process, on which it supervenes, and on the other hand, on the mind, since C is assumed, in the framework of GFO, to be a cognitive construction. One of GFO’s unique selling features is the integration of continuants, processes, and presentials into a uniform system. Hence, GFO integrates a 3D-ontology and a 4D-ontology into one coherent framework. Details of this integrative ontology are expounded in [14], [15].

#### Situoids and situations

Situoids are temporally extended parts of the world which can be comprehended as a whole. An example is a football match, happening in time, and including all necessary participating entities, among them the players, the football, the goal and other entities, but also the localization and the corresponding environment. The notion of “comprehended as a whole” is used here in an informal manner. We consider this notion as primitive which cannot by defined by other notions, and, hence, it must be characterized by axioms. A situation can be understood as a snapshot of a situoid, hence a situation is a part of the world, which is located at a time point, and which can be comprehended as a whole. Situations and situoids are considered as individuals, though, for their specification universals, in particular relational universals, are associated to them. Atomic constituents of situoids and situations are called facts. The specification of facts needs relations, relators, and roles. A relation is a category whose instances are relators. With relations, relators and roles, all components of facts are available, such that a more formal approach can be established. Since relations are entities connecting others, it is useful to consider collections of entities and their relators. The simplest combinations of relators and relata are *facts*. Facts are considered as parts of the world, as entities *sui generis*, for example “John’s being an instance of the universal Human” or “the book B’s localization next to the book C” refer to facts.

The ontology of situoids and situations, expounded in GFO, relies partially on the situation theory of [20] and has a root in the early philosophy of L. Wittgenstein [21].

#### Attributives, bearers and properties

Attributives are individuals, which are connected to other entities, called bearers. There are a variety of types of attributives, among them, qualities, roles, dispositions, functions, and structural features. The bearers of these attributives can be continuants, presentials and processes. But also attributives themselves may be bearers of attributives. Categories whose instances are attributives are called properties. According to the different types of attributives (relational roles, qualities, functions, structural features etc.), we distinguish quality properties (or intrinsic properties) and role properties (extrinsic properties), and the role properties are classified into relational role properties (abr. relational properties), social role properties (social properties) etc. [15], [22]. We take up the approach by Hoehndorf in [23], and equip a property P with a relation R and a category Q, such that the instances of P are connected by R with the instances of Q. Hence, in this framework, a full specification of a property P is given by a triple (P,R,Q).

#### Categories

In contrast to other top-level ontologies, for example, DOLCE (Descriptive Ontology for Linguistic and Cognitive Engineering [24]) or BFO (Basic Formal Ontology [25]), the ontology GFO provides an ontology for categories. We distinguish at least three kinds of categories: universals, concepts, and symbol structures. We hold, that any fully developed foundational ontology must include these three types of categories. *Universals* are constituents of the real world, they are associated to invariants of the spatio-temporal real world, they are something abstract that is in real things. Concepts are categories that are expressed by linguistic expressions and which are represented as meanings in someone’s mind. *Symbols* are signs or texts that can be instantiated by tokens. There is a close relation between these three kinds of categories: a universal is captured by a concept which is individually grasped by a mental representation, and the concept and its representation is denoted by a symbol structure, being an expression of a language. Texts and symbolic structures may be communicated by their instances that are physical tokens.

Besides this basic classification of categories, GFO provides categories of higher order by ascribing structural types to them. A more detailed theory of structural types of entities is expounded in [15]; in the current paper we restrict to those structural types, which are denoted by natural numbers, 0, 1, 2, ... Such order types for entities are inductively defined.

Every individual has order 0, and every entity of type greater than 0 is a category. A category is of order 1 if all of its instances are of order 0. A category is of order n+1 if all of its instances are of order n. Hence, the category *dog* is of order 1, whereas the category *species* is of order 2, since its instances, for example *Dog*, *African Elephant*, *Chimpanzee*, *..*, are categories of order 1. In section *Metadata and application data* we need entities of different orders to achieve an adequate semantic basis. In table 1 relevant entities of GFO are summarized.

**Table 1:** Selected GFO notions utilized in the current paper

Symbol	Name	Description/Definition	Example
*Cat*(*x*)	Category	*x* is a category, an instantiable entity, independent of time and space	The notion of ape, without further specification to concept. universal, or symbol structure

*Conc*(*x*)	x is a concept	x is a concept, an instantiable abstract entity which has a representation in the mind	A particular type of category. The concept of ape is grasped by the mind by a prototypical representation.

*Univ*(*x*)	x is a universal	x is an abstract, instantiable entity, existing independent of the mind, is in the real things	A particular type of category. The universal “Ape” is some invariant of reality, Aristotelian category

*Symb*(*x*)	x is a symbol structure	x is abstract, instantiable entity, whose instances are tokens	The abstract letter *A* whose instances are individual characters written or printed on a sheet of paper.

*Ind*(*x*)	Individual	x is a non-instantiable entity.	*x* can be concrete: *this car*, or abstract: *the uniquely determined number* π

*Cont*(*x*)	x is a Continuant	*x* is persisting individual exhibiting at time points wholly presented objects	*This ball*, persisting through time, and having a lifetime

*Pres*(*x*)	x is a Presential	x is an individual, being wholly present at a time point. A snapshot of a continuant.	*This ball at a certain time point t*; a snapshot of the continuant "ball".

*Proc*(*x*)	x is a Process	Temporally extended entity, happening in time.	This surgical intervention, with a certain temporal extension, and the surgeon, the patient and other persons as participants.

*Sit*(*x*)	x is a situation	x is type of whole existing at a time point, a part of the world, present at a time point which can be comprehended as a whole.	The snapshot of a lecture, including the snapshots of the lecturer, of the participants, the tables, the blackboard, and other entities, that allow to grasp this part of the world as a coherent whole at a certain time point.

*Situ*(*x*)	x is a Situoid	x is a temporally extended coherent part of the world that can be comprehended as a whole. It is a processual counterpart of a situation.	The course of a lecture at a certain location, during a certain time interval, and including the lecturer, the participants, the tables, blackboard, and other entities, that allow to grasp this part of the world as a coherent whole.

*Rel*(*x*)	x is a relation	x is a category, called relation, whose instances are relators	The father relation VR. An instance of VR is a relator R, being an individual. with two parts: the father role, and the child role.

*Relator*(*x*)	x is a relator	x is an cognitive entity, connecting players who play roles, being parts of x	John is father of Mary. There is a relator r, being an instance of the relation VR, r has two parts, being roles: the father role, played by John, and the child role, played by Mary

*Role*(*x*)	x is as role	x is a part of a relator, being an instance of a relation	The father role, played by John, father role is a part of a relator, being an instance of the relation VR

*Fact*(*x*)	x is a fact	x is an atomic constituant of a situation or situoid	John’s looking at the blackboard (is a constituant of a course of a lecture at a certain location)

*Attr*(*x*)	x is an attributive	x is an individual characteristics, trait, or feature, possessed by a bearer	This red *r* of this apple *a*.

*Prop*(*x*)	x is a property	x is an abstract and instantiable counterpart of an attributive	The abstract colour red, whose instances are individual reds inhering in bearers.

*instance_of*(*x*,*y*)	Instantiation x is instance of y	*x* is an instance of category *y.* (this is a primitive relation)	This ape is an instance of the category Ape

*part_of*(*x*,*y*)	x is part of y	x is a part of the entity y (this is primitive relation)	An arm is a part of a human body

*has_attr*(*x*,*y*)	x has attribute y	x has/possesses the attributive y (this is a primitive relation)	This apple x is the bearer of this red y, being an instance of the colour *red.*

*has_prop*(*x*,*y*)	x has the property y	x has the property y (this is a primitive relation)	This apple x has the colour red y means that there is an instance of the property colour red that inheres in this apple.

### Outline of the ISO/IEC 11179 standard

The *International Organization for Standardization* (acronym ISO) develops and provides standards to achieve desirable characteristics of products and services, among them, quality, safety, reliability, efficiency and interchangeability [6]. A standard for a domain can be understood as a unified, and generally accepted system of rules, conditions and definitions whose usage supports the afore-mentioned desirable characteristics for the entities of the domain. Any standard includes a system of basic notions which are used to explicitly specify the rules, conditions, and the definitions. The standard, considered in this paper, addresses the semantics and representation of data and information.

#### Overview

The objectives of the ISO/IEC 11179 Standard are described in [7] as follows:

“ISO/IEC 11179 - Metadata registries (MDR), addresses the semantics of data, the representation of data, and the registration of the descriptions of that data. It is through these descriptions that an accurate understanding of the semantics and a useful depiction of the data are found.

The purposes of ISO/IEC 11179 are to promote the following:

• Standard description of data

• Common understanding of data across organizational elements and between organizations

• Re-use and standardization of data over time, space, and applications

• Harmonization and standardization of data within an organization and across organizations

• Management of the components of data

• Re-use of the components of data”

According to [7], the ISO/IEC 11179 is a general description framework for data of any kind, in any organization, and for any purpose, independent of the application or subject matter area. It provides a model for a Meta Data Repository (MDR), which is designed to capture all the basic constituents of the semantics of data. The ISO/IEC 11179 specifies the kinds of metadata, necessary to describe data, relationships between them and the management and administration of that metadata in a MDR, such that the metadata can be shared among people and machines. The content of the ISO standard (abbr. of ISO 11179) is presented in the framework of a formalism, using UML diagrams and natural language definitions and descriptions.

The ISO standard proposes a general representation schema for data and information of any kind, and hence tries to achieve completeness in the sense, that any type of data can be adequately modelled in this framework. We do not believe that this schema is complete and universal, but instead we understand it as a stage of an evolution, which is directed to such an ideal complete data representation system whose achievement is an open problem. Top-level ontologies are aimed at the solution of a similar task, which pertains to the adequate modelling of arbitrary entities of the world.

#### Basic elements of the ISO/IEC 11179 standard

In this section we outline the basic elements of the ISO/IEC 11179 standard [7], [8]. The most basic entities are called *data element*, *data element concept*, *conceptual domain* and *value domain*. We review the definitions of the main entities as expounded in the standard.

A data element (DE) contains two main parts: a semantic one, called data element concept, abbreviated by DEC, and a representational one, called value domain and denoted by VD. A DEC may be separated into two components: the object class, which is understood to be a set of ideas, abstractions, or things in the real world that can be identified with explicit boundaries and meaning and whose properties and behavior follow the same rules; and a characteristic, which can be attributed to the members of the object class. A data element concept will be associated with exact one conceptual domain, denoted by CD. Conceptual domains come in two (non-exclusive) subtypes. An enumerated conceptual domain is specified as a list of value meanings. A value meaning is the semantic content (the meaning) of a value being a sign. Hence, a value designates its value meaning. A described conceptual domain is specified by a description. Conceptual domains will be represented by value domains. A value domain for a data element is a set of permissible values which exhibits a mapping between value meanings (elements of the representing CD) and values designating these value meanings.

## Methods

In this section we summarize the methods, being used to achieve an ontological foundation, and hence, a well-established semantic basis for the ISO standard, that is outlined in the preceding section *Outline of the ISO/IEC 11179 standard*. The usage of GFO is a part of our method, whose basic idea consists in the reconstruction and modelling of the entities of the ISO standard within the framework, provided by GFO. This generic method of reconstruction, or of modelling the entities of a domain D within the framework of a top-level ontology was introduced in [9], [13], [12], and called the *method of ontological reduction*. Throughout the paper, we use the expressions *ontological reduction*, *ontological modelling*, or *ontological analysis* as semantically equivalent phrases. There are subtle distinctions between these expressions, though, for the purpose of the current paper these refinements must not be taken into consideration.

(1) General method of ontological reduction (ontological modelling, ontological analysis)

The realization of this method assumes the usage of a top-level ontology, denoted by TLO. Let X be a set of entities, notably terms, denoting categories or relations, or even individuals, all of them are associated to a certain domain D. We want to specify, to completely explain the entities of X by using only those entities which are provided by the top-level ontology TLO. This can be made explicit as follows. The ontology TLO is specified by a system of categories, and relations, whose symbolic representations are captured by a vocabulary, and a set of axioms, describing the meaning of the vocabulary implicitly. Hence, TLO can be represented as a triple TLO = (L, V, Ax(V)), where L is a formal language that provides the means and procedures to describe content by formal expressions of L. An entity E of X is successfully reconstructed or analyzed by the expression Expr of L if the meaning of E is equivalent to the meaning of the expression Expr, relatively to the axioms Ax(TLO).

For example, to reconstruct, say, the category “organism”, formally denoted by Org(x), the desired expressions could have a form of “Org(x) iff x is a material structure with a closed natural boundary, and which is composed of subcomponents and interactions such that this material structure yields an autopoietic system”. The notions of material structure, natural boundary, subcomponent (using the part_of relation), and autopoietic system must provided by the top-level ontology TLO [26]. A top-level ontology which is complete for the principal notions of a domain D is called a core ontology for D. Usually, a top-level ontology, which includes only the most general categories of the world, is not sufficient to achieve a full ontological reduction of the domain’s entities. In this case, the ontology TLO must be extended, by adding new notions, relations, and axioms. The construction of such extensions, starting from a top-level ontology TLO, can be carried out by three basic steps, called in [9]: (a) adding a set of new primitive concepts and relations (extension step), (b) adding new axioms to implicitly describe the meaning of the new primitive concepts and relations (axiomatization step), (c) constructing a definition within the extended ontology (definability step). A more detailed description of this procedure is expounded in [9]. For the purpose of the present paper we need only the basic ideas to explain the main results in section *Results*.

(2) Suitability of GFO

We overview those basic features of GFO that demonstrate GFO’s suitability for the ontological analysis of the ISO standard, and for the domain of clinical trials in general. GFO provides an ontology of categories with two basic features, the availability of three basic types of categories (Universals, Concepts, and Symbolic Structures), and the introduction of categories of higher order. We show in section *Results* that this ontology of categories is needed to achieve an accurate modeling of ISO’s basic entities.

Another important feature of GFO is the ontology of properties and attributives, which is inspired by ideas of R. Hoehndorf, expounded in [23]. This approach is summarized in section *Basics of GFO*. In ISO, a property, called in ISO characteristics, is a constituent of the data element concept. A property is related to an object class, being another constituent of the data element concept. A further constituent of a data element is the conceptual domain, which arises from a classification of the instances of the property, in many cases by introducing scales, based on measurements. Then, there is the representational component of the data element, called value domain. The values of the value domain correspond to the notion of token of GFO. Tokens are instances of symbolic structures, being a particular category type in GFO. If we consider the semantic part of a data element, we may – in the framework of GFO – characterize what the instances are. The instances are facts, being parts of the real world, and on the representational side these facts are denoted/represented by infons, which can be considered as elementary propositions. For example F: “John, having the weight of 80 kg” describe a fact and the proposition P: ”John has the weight of 80 kg”, then, P is satisfied by the fact F. To achieve a complete description of the ISO entities, we need several kinds of categories, in particular concepts and symbolic structures, and we need an expressive basis for capturing properties and their bearers.

The Integration of objects and processes is another basic feature of GFO that is of particular importance for modeling of clinical trials. A clinical trial has a relation to a very complex process, which exhibits many subprocesses at different levels of granularity. On the most general level, we may distinguish the subprocesses of planning, execution, and analysis (evaluation, interpretation), on a lower level we may find such processes as “measurement of blood pressure”, or the course of a disease. Furthermore, in all these processes various entities participate, for example physicians, probands, blood traits and many others (the used instruments, for example). The modeling of such complex entities needs an expressive ontological framework, allowing an integration of processes and objects. GFO has the unique selling feature to provide an integration of these entities into one uniform system. GFO was already successfully applied to the field or surgical interventions, whose modeling needs an integration of objects and processes [27].

(3) The specification language of GFO

The description and ontological reconstruction of ISO’s categories must be carried out within a suitable formal language. We use FOL (First Order Language [28], [29]) as the basic formalism, and, additionally, other formalisms as, for example, UML (Unified Modeling Language [30]), but also OWL (Web Ontology Language [31]). The categories of individuals can be formally represented by unary predicates within FOL. On the other hand, we use categories whose instances are categories, hence, we admit higher order categories, whose adequate formalization needs higher order logics. FOL does not allow for a quantification of relations and higher order entities. For the usage of a formal language with sufficient expressive power there are several options. One of them is the use of Common Logic (CL [32]) which is a type-free first order logic, that implicitly allows the specification of entities of higher order, similar as ZF (Zermelo Fraenkel Set theory [33]), being a first order set theory without types. The weakness of CL, in our opinion, is the lack of a clear semantic basis and its formal axiomatization.

Another option is the use of a higher order logic, which must be adapted to a language of categories of higher order. The development of a suitable uniform formal language for GFO is work in progress; at present a combination of different language types and formalisms is used.

## Results

The ISO standard [7], [8] claims that it provides a semantic framework for data, though, several notions introduced are lacking a sufficiently established semantic basis. Some definitions of the ISO standard are contradictory or imprecisely specified. The notion of *metadata object* is defined, e.g., as an *object type*, *defined by the metamodel*, and the notion of *metadata item* is introduced as an *instance of a metadata object*. In our view, an object can neither be an object type, nor can it possess instances.

In the current section we analyse several notions of the ISO standard and show how they can be made explicit by using a deeper founded and more accurate semantic basis, which can be provided by an appropriate top-level ontology. In our opinion, GFO is especially suited for this purpose, since it allows, among others, for categories of higher order.

According to the ISO standard, we make a distinction between the model classes or objects, and the entities which they model. We also use the same notation, e.g., “Object_Class” for the model class and “object class” for the entity, modeled by the model class.

In the section *Metadata and application data* we examined the connections between these entity types, while in the section *Architecture of the metadata repository* we ontologically analyse the modeled real world entities. In section *Extensions of the ISO 11179* extensions of the ISO standard are considered.

### Metadata and application data

Metadata are data that define and describe other data. The ISO standard distinguishes between *metadata objects* and *metadata items*. According to the ISO standard, a metadata object is an entity which is defined by the metamodel. Examples of metadata objects include, among others, the entities Data_Element, Data_Element_Concept, and Permissible_Values. In the sequel, we call the metadata objects *metadata types* (table 2)*.* According to the ISO standard, *instances of metadata specify types of application level data*, whose instances, being *real world data*, are stored in the databases of the application level. Analogously to the metadata types and metadata items, we call the associated entities of the application level *application level data types* und *application level data items* (table 2). The entities, called *metadata type*, *metadata item*, *application level data type* and *application level data item*, do not belong to the data model, and hence, will not be implemented. In contrast to the *model level*, they belong to the *description level*, because they describe the data model entities as its instances.

**Table 2:** Describing entities, model entities, and real world entities and their order

ISO 11179 descriptive entity	ISO 11179 definition	Notation within the GFO framework	Example: model entity, being an instance of a descriptive entity	Real world entity, modeled by a model entity
***metadata object***	*is an object type*, *defined by metamodel*	***metadata type*** **(2)**	Classes **(1)**:*Object_Class;Characteristic*	Categories **(2)**:*Object class; Property*

***metadata item***	*is an instance of a metadata object*	***metadata item*** **(1)**	Objects **(0)**:*Person; Blood Pressure*	Categories **(1)**:*Person; Blood Pressure*
	
***type of application level data***	*is specified by a metadata item*	***application level data type*** **(2)**	Classes **(1)**:*Person; Blood Pressure*	

***real world data***	*is an instance of application level data type*	***application level data item*** **(1)**	Objects **(0)**:*John; Blood Pressure of John*	Individuals **(0)**:*John; Blood Pressure of John*

(x) - entity of order x

**Figure 1:**
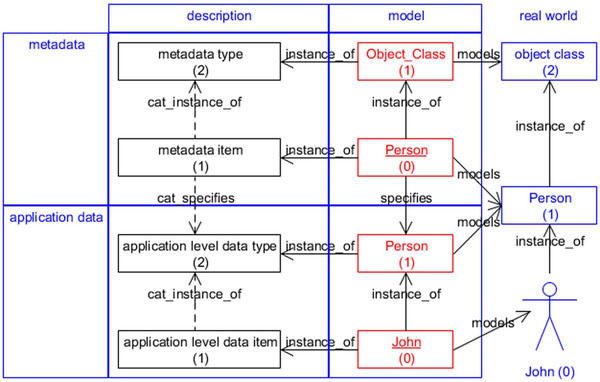
Metadata and application data

Within the framework of GFO, the considered entities have different orders. According to section *Basics of GFO*, entities of order zero are *individuals* and entities of order higher than zero are *categories*. On the descriptive level, the entity *metadata type* is of second order, whereas the entity *metadata item* is of first order. Its instances are model level entities, e.g. *Object_Class* and *Person*, accordingly. The entity *Object_Class* (model class) is of first order, whereas its instance, the entity *Person* (model object), is of the order zero. We demonstrate these relations by an example, displayed in figure 1.

Furthermore, the metadata item *Person* (model object, order zero) specifies the application level data type *Person* (model class, first order). The instance of the application level data type *Person* is a model object, called *John*, and is of order zero. The model object John is also an instance of the descriptive entity application level data item. To get a full picture, we introduce two relations between the categories of the description level, *categorial instantiation* and *categorial specification*. These relations can be defined, by using the usual instantiation relation, as follows:

*categorial_instance_of*(*x*,*y*) *:*= *∀a* (*instance_of*(*a*,*x*) *→ ∃b* (*instance_of*(*b*,*y*) *∧ instance_of*(*a*,*b*))*.*

*categorial_specifies*(*x*,*y*) *:*= *∀a* (*instance_of*(*a*,*x*) *→ ∃b* (*instance_of*(*b*,*y*) *∧ specifies*(*a*,*b*))*.*

The model entities model the real world entities. The entity Object_Class models the category *object class* which is of second order. Both the metadata item *Person* and the application level data type *Person* model the category *Person*, being of first order, and the application level data item *John* models the real world individual *John*. Between these three real world entities (of order two, one and zero) exists the instantiation relation respectively.

By using the expressive power of GFO, in particular the ontology of categories, we achieved an ontological reconstruction of the main notions of the ISO standard within the GFO framework.

### Architecture of the metadata repository

**Figure 2:**
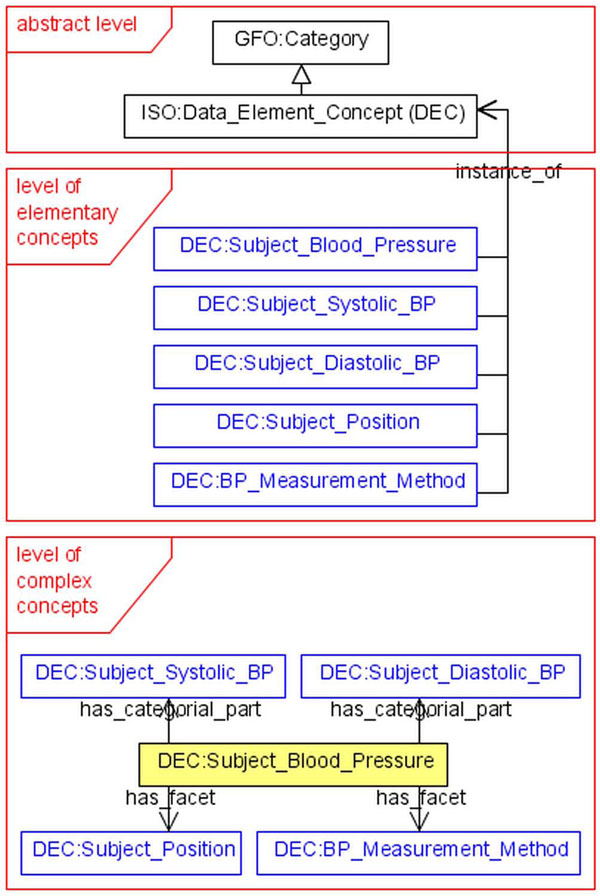
Architecture of the metadata repository

In the present paper we propose an architecture for a metadata repository, which is intended to be used for clinical and epidemiological research. The architecture of the Metadata Repository (MDR) consists of three levels with associated modules (figure 2).

The *abstract level* includes the ISO/IEC 11179 standard, but at the same time, it extends it and presents an ontological foundation by the top-level ontology GFO (section *Basics of GFO*). Furthermore, the notions of an item, of a property and others are ontologically analyzed and described at this level. This analysis is useful because it provides a semantic basis for the ISO standard which itself contains, from an ontological point of view, only a small fragment of the GFO framework. The module of the abstract level presents the metamodel. The *level of elementary concepts* presents the concepts associated to the items. These concepts are instances of the metamodel categories from the abstract level like data element concept, object class, characteristic (in GFO, the meaning of the term *characteristic* corresponds, roughly, to the meaning of the term *property*) or item itself. The third level is presented by the *level of complex concepts*. The complex concepts are constructed from elementary ones by connecting them by different relations.

Example (figure 2):

• *abstract level*: ISO:DEC is a subcategory of GFO:Category

• *level of elementary concepts*: Concrete DECs will be defined (as instances of ISO:DEC): DEC:Subject_Blood_Pressure, DEC:Subject_Systolic_BP,

• *level of complex concepts*: The concepts of the second level will be combined to complex concepts by various applicable relations (The complex concepts have components like categorial parts or facets). For example, the complex concept DEC:Subject_Blood_Pressure has two categorial parts und two facets.

Subsequently, we demonstrate our method by few examples; in particular, we investigate and analyze the notion of a data element in more detail. The whole/full metamodel exhibits an analysis of many other categories of the ISO standard, and provides further categories, which are not yet present in the standard.

**Figure 3:**
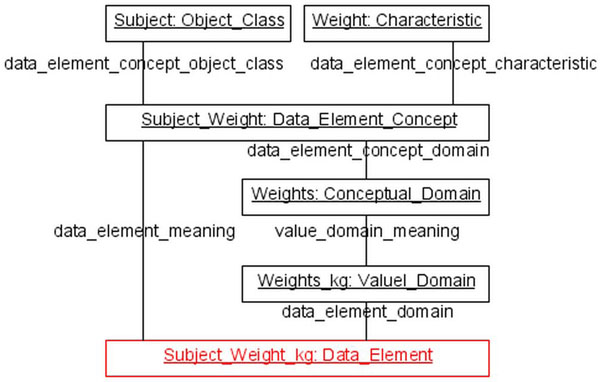
Structure of a data element

A data element (e.g. *subject’s weight in kg*) has two constituents/components, a semantic one, called data element concept (e.g. *subject’s weight*), and a representational one, called value domain (e.g. *weights in kg*) (figure 3).

We must clarify how its components relate to the whole. For this purpose we introduce a suitable part_of relation, called *constituent_part*. Then, data elements have certain constituents.

A data element concept includes an object class (e.g. *subject*) and a property (e.g. *weight*). An object class is a category whose instances are entities of the real world (e.g. individual subjects like *John*). The property, being a constituent of the data element concept, is a category whose instances are attributives (e.g. individual weights like *John’s weight*, see section *Basics of GFO*), which are connected to objects (the bearers), being instances of the object class. Every property P is equipped with a relation, which connects the instances of P with the bearers, being instances of the object class. In our example, the property *weight*, whose instances are qualities, is equipped with the inherence relation.

A uniquely determined conceptual domain is associated with a data element concept. This association is captured by the relation *has_conceptual_domain* (*DEC*, *CD*). The conceptual domain is a set of entities, called value meanings, which serve as possible property values. At this place we must clarify what property values are. Let us consider as an example the property weight, denoted by W. As described above, the instances of the property W are attributives (e.g. *John’s weight*). We may partition the instances of W by a measure, say, g, kg. Then, for example, 70 kg represents an equivalence class which exhibits the set of all instances of W which are measured as 70 kg. Hence, W(70kg) may be considered as a subproperty of W. But, the main point is that by using a measuring process we get a natural partition of the instances of W into equivalence classes (property values), which are the value meanings being included in the conceptual domain.

The instances of data element concepts are *facts*. A fact, e.g., *John’s weight being 70 kg* includes an instance of the object class (*John*), an instance of the property (*John’s weight*) and a value meaning (property value) from the conceptual domain (*70 kg*). Thus, data element concepts are categories (subcategory of GFO *category*).

**Figure 4:**
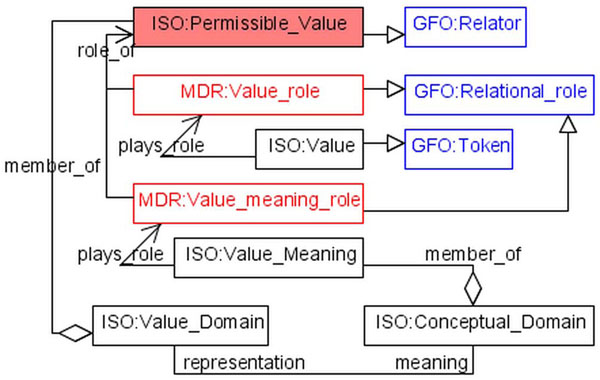
Permissible value relator

A conceptual domain can be represented by several value domains. A value domain is a set of permissible values consisting of value-meaning/value pairs. The relation between value meanings and values can be ontologically specified by using the notion of a *relator* of GFO. Relators are instances of relations with parts being called relational roles. We introduce a (value meaning, value)-relation, briefly denoted by R_mv_, whose instances are relators. The relators of R_mv_ are individuals with two parts, called roles, the value role and the meaning role. These roles inhere in the players, and the player of the meaning role is a member of the conceptual domain, and the value role is played by token. A token is considered in the current context as an instance of a symbol structure (figure 4).

Because the semantic constituent of data element (a data element concept) is a category, also the data element itself can be considered as a category. The instances of a data element are called *fact-representation*s. A fact-representation has two constituents: an instance of a data element concept, being a fact (see section *Basics of GFO* for an explanation), and a value from the value domain (token, representation).

The analysis of the notion of *item* (especially as it is used in the ZKS Leipzig -Zentrum für Klinische Studien [34]), yields the result that the category item (also denoted by the term *item category*) must be characterized within the category of data elements. Additionally to the properties of data elements, items have further properties (e.g. default, range or data acquisition). Data acquisition, for example, is a process (a subcategory of GFO category *process*) that is associated with each item (e.g. blood pressure measurement and blood pressure item). It has a temporal extension (time interval, in GFO a *chronoid*), different participants (subject, doctor, measuring instrument and a fact as a result of the measurement) and can be of different types (questioning, counting, measuring, observation). Hence, the category of data element must be intensionally extended to the item category. The item category can be considered as an extensional subcategory of a category of data elements.

**Figure 5:**
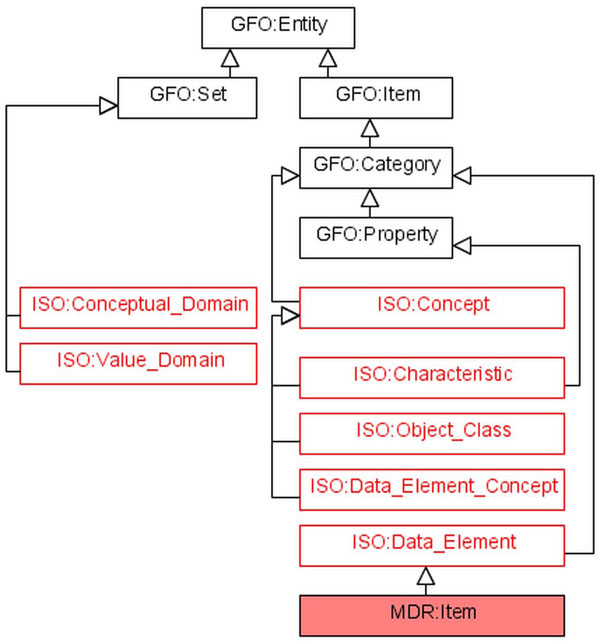
Ontological embedding of ISO standard in GFO (partial tree)

We summarize the considered entities in figure 5, displaying the ontological mapping into GFO. In this partial tree there are two kinds of extensions presented, in the upwards direction certain categories from GFO are included, for example, the categories GFO:Entity, GFO:Set, GFO:Item etc. In the downward direction a new category occurs, the *category of items*. As described above, a data element is a special category, and an item is a special data element.

### Extensions of the ISO 11179

In this section, we consider extensions of the ISO standard, which are based on the introduction of particular relations and additional classes. The ISO standard can be understood, from an ontological viewpoint, as a system of categories which are connected by certain relations. Such a system can be extended in two ways. We may add categories and relations on the level of the metamodel, or we may add instances of the metaclasses, notably of the metaclass *relation*. The former type of extension we call *horizontal extension*, the latter type is called *vertical extension*. We developed a horizontal extension of ISO, by adding a number of metaclasses, including, among others, *Item*, *Collections*, and *Range*, because they are relevant in applications to clinical trials.

Furthermore, we added a number of concrete relations, being instances of the metaclass *relation.* These additional relations are important to compose elementary properties to complex ones. The first relations are the relations categorial_part (X,Y) and has_facet (X,Y). The latter plays an important role for the definition of variants of items. There are further relations which are important for the composition of atomic properties to complex properties. These relations are established on the levels of individuals (on the level of attributives), and then lifted to the level of categories. These relations include part_of, has_function, and the ternary relation function_realizes (X,Y,Z), the function X is realized by the process Y with the realizer Z.

There is an important class of relations, connecting attributives with bearers. For example, the quality *red*, being an individual, inheres in this rose, being the bearer. Such relations can be lifted to the level of properties and bearer categories. This important idea was presented in [23], and leads to refined ontology of properties. Properties P are equipped with a relation R and a category Q, such that the instances of P are connected by R with instances of Q, being the bearers for P.

The combination of attributives, and hence, of properties, is a core aspect of the definition of complex phenotypes. The envisaged extension of the ISO standard will support the definition of complex phenotypes. The development of ontologies of complex phenotypes will use the work of PATO (Phenotypic Quality Ontology [35]), and the Human Phenotype Ontology [36].

### Implementation

Clinical research can be supported by the reuse of items. Such support assumes that items are captured and specified in a semantically correct way, and are computationally presented such that they can be efficiently retrieved and used. For this purpose we implemented a software prototype whose first version allows to create and to manage all fundamental concepts of the ISO standard such as conceptual domain, value domain, data element concept, data element and so on. Moreover, some of our extensions of the standard like items, item groups and item variants were implemented. The graphical representation of the concepts is also possible. But the most important function is that the simple concepts can be connected by suitable relations to complex ones (see *level of complex concepts* in figure 2). These relations can come, e.g., from top-level ontologies (in our case from GFO).

We decided to continue the implementation of the prototype by adding new functionalities to be used it in future clinical and epidemiological studies. Also we collect documentation features of the LIFE project (Leipzig Research Center for Civilization Diseases [37]) to fill the repository of data. Last but not least, the prototype is also used to test our metamodel. The software is web-based and uses a relational database. The first version is developed in PHP with MySQL database connection.

## Discussion

### Semantic integration of data

In this section we consider the second use case that pertains to the problem of information integration and interoperability between information systems. There exist a variety of information models in the field of health care, including, among others, HL7-RIM (Reference Information Model [38]) and OpenEHR (Electronic Health Records [39]). These models are used in different institutions, and, obviously, the realization of the interoperability between them is an important task. We sketch a solution of this problem by using the method of ontological analysis set forth in the current paper. In the first step, we analyze the information models HL7-RIM and OpenEHR in the framework of GFO. The entities of the information models (called, for example, *Entry*, *Act*, *Observation*) are instances of the descriptive entity *application level data type* (s. section *Metadata and application data*). They model the real world entities of first order, whose instances are individuals. Hence, these real world entities are subcategories of the first order entity *individual* of GFO.

On the other hand, the basic metadata types of ISO 11179 (Data_Element, Object_Class ect.) model the real world entities of second order (data element, object class ect.). They are extensional subcategories of the second order entity *category* of GFO. Hence, the entities of ISO 11179 and those of the information systems as well as the entities, which are modeled by them, can be uniformly embedded in the categorial system of GFO. We now use the relation *categorial instance* (introduced in section *Metadata and application data*) which relates entities, modeled by the information systems to those, modeled by the ISO standard. For example, the entity *observation* can be understood as a categorial instance of the entity *data element*. This would imply that, in this example, every instance of the entity observation, for example the *blood pressure of John on 01.01.2001*, should be an instance of a suitable concrete data element, say, the data element *blood pressure*.

**Figure 6:**
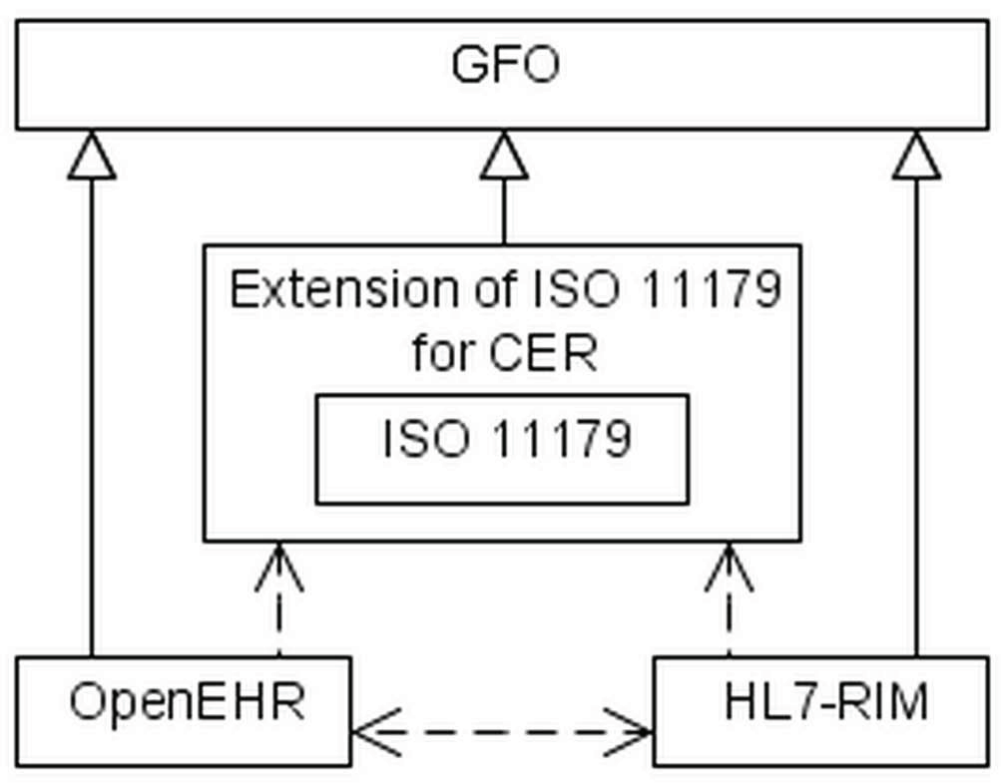
**Semantic integration and interoperability. ** CER – clinical and epidemiological research.

The semantic integration of different information models within an ontological framework, as GFO and the ISO standard, enables the realization of the interoperability between the information systems implementing those models (figure 6). The complete semantic integration of the information models of OpenEHR and HL7-RIM will be published in a separate paper.

### Application to clinical trials

The methods and tools, outlined and discussed in this paper, are intended to have two main applications in the context of clinical trials. The first application pertains to the planning and realization phase of clinical trials, the second application is intended to support the interpretation phase of a clinical trial.

Every clinical trial needs an accurate specification of the data, to be acquired and evaluated, and of the characterizing metadata. Data in clinical trials arise as a result of so-called data source processes by e.g. questionnaires, interviews, physical examinations etc. These include a description of the data to be collected, such as format, prompt, etc. These metadata that we designated in the context of clinical trials items or documentation features, support a variety of functions. A well-established ontology of items can support the work of the study designer and also of the data manager. A computer-based tool of the kind of our prototype can help to design a study, in particular, to support the decision about the selection of data and of the choice of the data-acquisition process. Such a tool can be used to develop a repository of items which can be managed by using the tool’s functionalities, among them, the search for items and the comparison between items. And it can also be used to assist the creation of new items. We envisage also a tool for the automatic generation of basic case report forms (CRFs). Also a tool can be possibly used to export items or groups of items, for example, as ODM (Operational Data Model of CDISC [40]), or in SQL for use in study management software or to create instance databases.

A second useful application is intended to support the interpretation phase of a study. An ontology of items can be used for annotation of data such that a researcher may identify and access those data, which are relevant for a current research question. It can be possibly used, to provide the appropriate instance data.

### Data elements, properties, and phenotypes

Recently, an international Human Phenome Project was proposed, and an overview on basic tasks and problems of this research programme was presented in [41]. These problems and tasks are of increasing importance because a main goal of genetic research is to identify genotypes that are related to human phenotypes. In particular, phenotypic information is relevant for the understanding of mechanisms that causes diseases. Research in this field needs a precisely defined and well-established notion of a phenotype. The authors in [42] notice, that inconsistencies occur in the usage of this term between various authors, and that this notion is insufficiently established. In this section we analyse the notion of a phenotype and relate it to the category of properties in GFO, and to the data elements of the ISO standard. Our investigation relies on the approach to phenotypes, as presented by Hoehndorf in [23]. We outline a formal ontological framework, based on GFO, to treat phenotypes, properties and data elements in a uniform manner.

Usually, a phenotype is considered as an observable characteristic or trait of an organism, such as its morphology, its biochemical or physiological properties, its function, or its behaviour [23], [42]. A phenotype can be considered as an individual, for example, *this blue of this eye*, then the term *blue* denotes a GFO attributive, or as an abstract instantiable entity, for example, *this eye has the property blue*, then the term *blue* denotes a category, possessing individual blues as instances. To make this distinction precise, we use the term *phenotype* for the attributive interpretation (in [23] these are called phenes), that is, to denote concrete spatial-temporal individuals, and the term *phenotypic property* to denote abstract entities, that is, properties. Note, that only spatial-temporal individuals can be observed, since an observation is a spatial-temporal process.

According to GFO, attributives and properties are dependent entities, they depend on bearers. Hence, there is an ontological basic relation, depends_on(x,y), expressing that the attributive x depends on the attributive y, which can be lifted to the abstract level prop_depends(X,Y), being a relation between properties X and Y. The relation prop_depends(X,Y) can be defined by the relation depend(x,y) and the instantiation relation instance_of(x,X), by the following condition: Every instance of X depends on an instance of Y.

Phenotypic properties can be further classified with respect to the type of the dependency relation, and as explained in section *Basics of GFO*, a complete specification of a property should additionally include a description of this connecting relation. For this purpose we introduce the following expression “X depends on Y via the relation R” which can be represented also as a triple (X, Y, R), where X is a property, Y is a category of bearers, and R is the connecting dependency relation. We preliminarily call such triples *property nexus* and propose to use the term *phenotypic property nexus* to denote those property nexus whose property component is a phenotypic property. A phenotypic property P itself (without the additional term “nexus”) can be considered as a phenotypic property nexus too, because it can be represented by the triple (P, Q, prop_dependent), assuming that - within a context - the bearer category Q is determined.

The fragment of the upper level ontology of phenotypes, as set forth in [23] and called upper level ontology of phenes, can be more accurately interpreted as a taxonomy of phenotypic property nexus. We consider several examples, inspired by [23], and present an interpretation within our framework. A qualitative phenotype is considered as an attributive related to a bearer by the inherence relation inheres_in (x,y). If we consider a quality-property, say Red, then we will get the following property nexus (Red, RedOb, inheres_in), where Red is the (abstract) property Red, RedOb is the category of red objects, and inheres_in connects the instances of Red, being individual reds; with the instances of RedOb.

Structural phenotypes are based, according to [23], on the mereological relation part_of.

A heart, for example, is a part of a human body, which yields the following phenotypic property nexus (Heart, HB, part_of), where every instance of the category Heart, being an individual, is a part of a instance of HB, being the category of human bodies. More intricate examples are related to processes. Let us consider the endocrine pancreatic cells, being involved in the insulin production process, denoted IPP. Then the following phenotypic property nexus might be specified (EPC, IPP, participates_in), where every instance of EPC participates in a process, being an instance of the category IPP.

We now turn to the data elements. Any data element has a data element concept as a constituent, and any data element concept consists of an object class OBC, and a characteristic, which can be understood as a property in GFO. It is tacitly assumed that the property depends on the object class OBC which itself is a category within GFO. The ISO standard does not specify what type of category can be assumed for OBC. We restrict the category OBC, in what follows, to be simple and concrete, hence, the instances of OBC are spatial-temporal individuals. The entity, which links phenotypes with data elements, is the property, being a constituent of the data element concept. Data elements do not provide the notion of a property nexus, since the dependency relation is missing, which connects the property with the object class. On the other hand, data elements provide the conceptual domain, which yields a classification of the instances of the property into classes. These classes ontologically correspond to the values of properties, as expounded in GFO and called in the ISO standard value meanings (s. section *Architecture of the metadata repository*). Theses property values arise, in many cases, from measurements, which exhibit an important dimension in practical applications, in particular, in the context of clinical trials. Furthermore, data elements provides other important constituents, notably value domains, which serve as representational means, which link the semantics with a symbolic level.

We may conclude, that the framework of data elements may be refined to include phenotypic property nexus.

## Conclusion and future research

We outlined basic ideas and results about an ontologically founded architecture for information systems, to be applied in the field of clinical and epidemiological research.

The information systems, based on this architecture, are usually related to particular domains, or classes of domains, and are aimed at a variety of applications. What is common to all of these information systems is the basic functionality to capture, structure, present and retrieve metadata, which are associated to a domain of interest. The ISO 11179 standard is a valuable initial system for such a basic architecture, though, the semantics of this standard is insufficiently established. According to our approach, we used a top-level ontology - in the present paper the ontology GFO - to elaborate an ontological foundation which establishes a more precise and fine-grained semantic framework for the ISO standard. Hence, in this way we established a bridge between the ISO standard and the rigorous methods of formal ontology. The ideas of this paper can be applied to arbitrary standards, and were, actually, already partially used in [43] for the semantic underpinning of the Unified Modelling Language (UML). Finally, we implemented a prototype for this architecture which realizes the functionality to capture and present items, and to retrieve items taken from clinical studies, which were captured and stored in the system. The development of this tool will be continued to include more functions, for example, reasoning capabilities.

There are a number of promising open problems and tasks for further research. One of the most important tasks is the ontological analysis of the notion of phenotype and related concepts, as phene, phenome, and canonical phenotype. The GFO foundation of ISO, augmented by refinements, as, for example, property nexus (discussed in section *Data elements*, *properties*, *and phenotypes*), might provide an expressive framework for the development and representation of phenotype ontologies, playing a role in clinical research.

The envisaged information systems may present ontologies of phenotypes for various fields of clinical research.

## Competing interests

The authors declare that they have no competing interests.
